# Herd prevalence of bovine brucellosis and analysis of risk factors in cattle in urban and peri-urban areas of the Kampala economic zone, Uganda

**DOI:** 10.1186/1746-6148-7-60

**Published:** 2011-10-18

**Authors:** Kohei Makita, Eric M Fèvre, Charles Waiswa, Mark C Eisler, Michael Thrusfield, Susan C Welburn

**Affiliations:** 1 Centre for Infectious Diseases, Division of Pathway Medicine, School of Biomedical Science, College of Medicine and Veterinary Medicine, The University of Edinburgh, 1 Summerhall Square, Edinburgh, EH9 1QH, UK; 2 Centre for Infectious Diseases, Ashworth Laboratories, The University of Edinburgh, Kings Buildings, Edinburgh, EH9 3JT, UK; 3 Faculty of Veterinary Medicine, Makerere University, P.O. Box 7062, Kampala, Uganda; 4 Veterinary Clinical Sciences, Royal Dick School of Veterinary Studies, College of Medicine and Veterinary Medicine, The University of Edinburgh. EH25 9RG, UK; 5 Current Address: School of Veterinary Medicine, Rakuno Gakuen University, Ebetsu, 069-8501, Japan, and Improving Marketing Opportunities Theme, International Livestock Research Institute, PO Box 30709, Nairobi, Kenya

## Abstract

**Background:**

Human brucellosis has been found to be prevalent in the urban areas of Kampala, the capital city of Uganda. A cross-sectional study was designed to generate precise information on the prevalence of brucellosis in cattle and risk factors for the disease in its urban and peri-urban dairy farming systems.

**Results:**

The adjusted herd prevalence of brucellosis was 6.5% (11/177, 95% CI: 3.6%-10.0%) and the adjusted individual animal prevalence was 5.0% (21/423, 95% CI: 2.7% - 9.3%) based on diagnosis using commercial kits of the competitive enzyme-linked immunosorbent assay (CELISA) for *Brucella abortus *antibodies. Mean within-herd prevalence was found to be 25.9% (95% CI: 9.7% - 53.1%) and brucellosis prevalence in an infected herd ranged from 9.1% to 50%. A risk factor could not be identified at the animal level but two risk factors were identified at the herd level: large herd size and history of abortion. The mean number of milking cows in a free-grazing herd (5.0) was significantly larger than a herd with a movement restricted (1.7, p < 0.001).

**Conclusions:**

Vaccination should be targeted at commercial large-scale farms with free-grazing farming to control brucellosis in cattle in and around Kampala city.

## Background

Brucellosis remains one of the world's most widespread zoonoses [1]. The disease in humans, known as 'undulant fever', 'Mediterranean fever' or 'Malta fever' [2], remains an important public health problem. On genetic grounds the *Brucella *grounp can be regarded as variants of a single species, *Brucella melitensis*, however for practical reasons, six main species are distinguished: *B. abortus*, *B. suis*, *B. melitensis*, *B. neotomae*, *B. ovis *and *B. canis *[2]. Historically, only *B. abortus*, *B. suis *and *B. melitensis *have been considered as zoonotic pathogens but recent reports have shown that the newly recognised marine mammal species also have zoonotic potential [3,4]. Out of these zoonotic *Brucella *species, bulk of human diseases is caused by *B. abortus *and *B. melitensis *[4]. Brucellosis in cattle (mainly caused by *Brucella abortus*) poses not only a significant threat as a source of infection to humans but also the risk of economic losses. Losses through abortion or calf death is a huge economic constraint for farmers [5] and establishment of the carrier state in a large proportion of animals may lead to a 20% reduction in the milk yield [6]. In areas where culling or other means of brucellosis control are not practised, long-term chronic infections are often associated with carpal hygromas and infertility [7]. Aborted foetuses and discharges contain large numbers of infectious organisms, and chronically infected cattle can shed lower numbers of organisms via milk and reproductive tract discharges, and can also vertically transmit infection to subsequently born calves, thereby maintaining disease transmission [7].

Human brucellosis has been found to be prevalent in urban areas of Kampala, Uganda [8]. The sources of the risks from informally-marketed milk and the effective control measures for human brucellosis have been described. Constructing boiling centres either in dairy production areas or peri-urban Kampala and enforcing traders to sell to these centres would reduce the risk the most [9]; however control of brucellosis in the source cattle would reduce the risks to humans most effectively. It is therefore important to know the prevalence and risk factors in cattle. A number of studies of brucellosis prevalence in cattle have been conducted in Uganda [10-15]. It has been suggested that the high plateau lands of western and eastern Uganda were zones of hyper-endemicity, for both human and bovine brucellosis, while the Central and Southern part of the Uganda along the shores of Lake Victoria were zones of moderate endemicity [16]. Brucellosis is also prevalent among the Ugandan wildlife population [17].

*B. melitensis *is normally associated with goats and sheep but can cause cross-species infection with dairy herds [2]. In eastern and western Uganda, 13% (12/93) of goat herds had goats with positive reaction in both the brucellosis card test and the *B. melitensis *tube agglutination test [18]; the risk of human and bovine brucellosis due to *B. melitensis *is not negligible in Kampala.

Recent studies in Uganda have shown that differences in disease prevalence in cattle are associated with different production systems [13,19]. In zero-grazing systems (for example, in Eastern Uganda) where there is a low level of herd-to-herd contact, the herd-level prevalence was low (5.5%) while in pastoral systems (for example, in Central Uganda) where there are high level of herd-to-herd contact, the prevalence was 100% [19]; this finding is supported across studies of brucellosis in sub-Saharan Africa [7].

The present study attempts to elucidate the prevalence of brucellosis, as well as risk factors for brucellosis in cattle in urban and peri-urban areas of Kampala, providing inputs for evidence-based disease control in Uganda.

## Results

### Prevalence of brucellosis

In total, 423 secondary sampling units (cows) in 177 (out of 625) primary sampling units (cattle herds) were sampled; the actual sample fraction at herd level was 28.3% (177/625). Out of 177 sampled herds, 11 herds were found to be positive showing an adjusted herd-level prevalence of brucellosis of 6.5% (95% CI: 3.6-10.0). At the individual animal level, 21 of 423 cows were positive with the competitive enzyme-linked immunosorbent assay (CELISA) and the adjusted prevalence was 5.0% (95% CI: 2.7 - 9.3).

### Herd size

**Figure 1:**
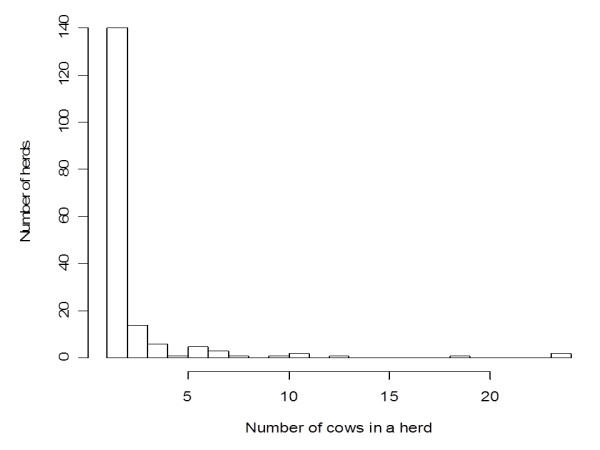
**The number of herds according to the number of milking cows in a herd**. The majority of the herds were small scale with one or two milking cows.

Figure 1 shows the frequency of herds according to the number of milking cows in a herd. The geometric mean [20] of number of cows per farm was 1.7. Six large herds with more than or equal to 10 milking cows were located in peri-urban and rural areas; however even in these areas, the majority of herds were small-scale, comprising less than 10 cows. The maximum herd size was 24 cows among all the 177 farms (a farm is equivalent to a herd in the present paper) and for the herds with less than or equal to 24 animals, the sample size in a herd was always calculated as all the cows belonged to; sera were collected from all the milking cows in the selected farms.

### Within herd prevalence

Table 1 shows the proportions of brucellosis positive cows according to the number of cows in a herd. Within herd prevalence of brucellosis among infected herds varied between 9.1 to 50.0%, and the herd level information-weighed overall mean within-herd prevalence was 25.9% (95% CI: 9.7% - 53.1%). There was no relationship between within-herd prevalence and herd size; the logit of within herd prevalence of brucellosis did not change with the number of milking cows in a herd (slope = -0.019, se = 0.03, *p *= 0.652).

**Table 1:** The number of herds with infected cows and within-herd prevalence according to the number of cows in a herd

Number of cows in a herd	Number of herds with infected cows	Within-herd prevalence (%)
2	3	50 (50)*
4	1	25
5	1	20
6	1	16.7
7	1	14.3
11	1	9.1
13	1	15.4
19	1	15.8
24	1	25

*Mean and range ()

### Risk factors for brucellosis at the animal level

No significant risk factor for brucellosis was detected, by univariate analysis, at the animal level. Neither the use of a bull for insemination, nor a history of vaccination against brucellosis nor any history of abortion were found to be either significant risk or preventive factors for brucellosis infections at the animal level (all *p*-values of prevalence ratios [21,22] were more than 0.05, see Table 2). Furthermore, at the animal level, the mean rank of body condition score was also not found to be significantly different between CELISA positive (median = 3.0, average rank = 218.2) and negative cows (median = 3.0, average rank = 211.7, df = 1, *p *= 0.799). The mean parity was not significantly different between CELISA positive (2.76, 95%CI: 2.06 - 3.69) and negative cows (2.39, 95%CI: 2.24 - 2.56, *p *= 0.341) and the mean age was also not found to be significantly different between CELISA positive (5.53) and negative cows (5.20, *p *= 0.420).

**Table 2:** Univariate analysis for brucellosis at the animal level

Factors	Diseased animals	Healthy animals	Prevalence (%)	Prevalence ratio	*p*-value
Insemination					
Bull	18	310	5.5	1.74	0.434
AI	3	92	3.2		

Vaccination					
Vaccinated	5	60	7.7	1.72	0.345
Not vaccinated	16	342	4.5		

Abortion					
Aborted	3	42	6.7	1.40	0.479
Not aborted	18	360	4.8		

### Risk factor of brucellosis at the herd level

In the univariate analysis, large herd size was associated with sero-positive status of herds; the number of milking cows in a herd was significant larger in sero-positive herds (8.6) than sero-negative herds (2.0, *p *< 0.001). Three factors-free-grazing farming, a history of vaccination and a history of abortion - had *p*-values less than 0.2 [23] in prevalence ratios (see Table 3). There was no collinearity between each two of these four factors.

**Table 3:** Univariate analysis for brucellosis at the herd level

Factors	Infected herds	Healthy herds	Prevalence (%)	Prevalence ratio	*p*-value
Urbanicity					
Urban	4	50	7.4	*x*^2 ^= 0.59*	0.743
Peri-urban	2	47	4.1	df = 2	
Rural	5	69	6.8		

Free-grazing					
Free-grazing	7	26	21.1	6.15	<0.001
Restricted	4	140	2.8		

Breed					
Improved	4	57	6.6	*x*^2 ^= 0.47*	0.790
Cross	3	61	4.7	df = 2	
Indigenous	4	48	7.7		

Insemination					
Bull	8	121	6.2	0.90	1
AI	3	45	6.3		

Vaccination					
Vaccinated	2	7	22.2	3.76	0.10
Not vaccinated	9	159	5.4		

Abortion					
Aborted	4	21	16.0	3.06	0.052
Not aborted	7	145	4.6		

Bought-in cattle					
Yes	7	119	5.6	0.61	0.716
No	3	40	7.0		

Persistent fever					
Exist	1	16	5.9	0.86	1
Not exist	10	150	6.3		

* Likelihood ratio test result

Two factors: large herd size (OR: 1.3 (95% CI: 1.1-1.5), *p *< 0.001) and history of abortion (OR: 4.1 (1.0-17.6), *p *= 0.059, it remained because of the biological plausibility) remained in the final model and the model passed the goodness-of-fit test, Hosmer-Lemeshow test [24] (sum of square = 53.1, df = 167, *p *= 0.60). Although the factor, free-grazing farming (OR: 2.7, *p *= 0.2) did not remain, its removal changed the logit of herd size by 19.8% ((0.26-0.217)/0.217, data not shown in a table), and by a GLM with quasipoisson errors, the mean number of milking cows in a free-grazing herd (5.0) was significantly larger than a herd with the movement of cows restricted (1.7, p < 0.001). This suggested that a risk factor, being large herd, was associated with free-grazing of cattle herds.

## Discussion

In the present study, a brucellosis herd prevalence and individual animal prevalence were observed in urban and peri-urban areas of Kampala using a probability sampling framework and a highly sensitive and specific diagnostic test: the CELISA. A previous study of only 16 farms (one in Kampala, five in Mukono and 10 in Wakiso) in central and southern parts of Uganda reported a high prevalence of brucellosis at the herd level (56.3%, 9/16) and at animal level, 5.0% (19/383) using both the Rose Bengal Test (RBT) and serum agglutination test (SAT) [15]. In a study of 245 cattle serum samples from urban and peri-urban areas of Kampala, 42% were positive for antibodies against *Brucella *spp. using the slow serum tube agglutination test [14]. However, both the SAT and RBT are less specific than the CELISA [25] and the reported high prevalence(s) at herd level might be due to false-positive serum reactions (FPSR) and or due to bias in farm selection. False-positive serum reactions in *Brucella *spp. screening tests are known to be caused by unrelated Enterobacteriaceae [26-35] and CELISA can eliminate such reactions [33]. *B. abortus *vaccination strain 19 also gives rise to an antibody response similar to that resulting from natural infection [33] but CELISA can eliminate this false-positive reaction only by approximately 50% [36]. In our study, 9/177 (5.1%) of farms held vaccinated cattle and the CELISA positive reactions of vaccinated cows in two farms might be due to the false-positive reactions with *B. abortus *vaccination strain S19.

The present study identified two risk factors for brucellosis at the herd level: large herd size and history of abortion. For the correct causality, the result should be interpreted that *Brucella *sero-positive status of herds was a risk factor of abortion. Free-range farming did not remain in the final multivariable model; however being a large herd was associated with a free-range farming. Moreover, the moderate change of logit of the factor - large herd, by a removal of a factor - free-grazing from a model suggested a confounding to large herd by free-grazing which can maintain or enhance infection with brucellosis in a herd. Free-grazing farming and abortion have been previously identified as risk factors relating to pastoral systems [7,19,37]. The unique characteristic observed in peri-urban areas of Kampala was the presence of commercial large-scale free-grazing farming within a fenced zone, although most farms are small-scale. This commercialized management system, including milk sales and animal health inputs e.g. periodical insecticide spraying, is also observed in the Mbarara dairy production area and could be distinguished from zero-grazing systems (improved breed) and communal-grazing systems (local and cross breeds) which are dominant in peri-urban Kampala. These commercial herds rarely come into contact with any other herds because they are separated by a physical barrier (a fence) but the transmission of brucellosis can be maintained within each large herd while grazing.

In the present study, a history of bought-in cattle was not a risk factor for brucellosis, suggesting the endemic status of brucellosis in and around Kampala may be maintained indefinitely by low-level within herd transmission. Use of bulls was shown not to be a risk factor. It might be also because of the endemic status; however two zero-grazing herds using artificial insemination were found to be infected with brucellosis in this study. Purchase of infected animals or contamination of frozen semen with *Brucella *could not be ruled out [6].

No risk factors for brucellosis at the level of an individual animal were identified in the present study. In Mbarara, previous studies have shown that keeping exotic breeds [13] was a risk factor but this was not found to be a risk factor in the present study. Age of animal has also been shown to be a risk factor in other studies in Mbarara, Uganda [13] and also in Northern Ireland [38]. While parity and age were not identified as risk factors in the present study, this may be simply because all samples were taken only from milking cows which are usually more than two years old.

The level of urbanisation in this study was not a risk factor for brucellosis in Kampala but large herds with free-grazing farming (which are risk factors) are located in peri-urban areas, and the Ugandan Veterinary Authorities should consider these areas for practical control programmes in and around Kampala, with a special focus on large farms with free-grazing farming.

## Conclusions

The present study found three risk factors for brucellosis in cattle in the urban and peri-urban areas of Kampala: being large herd, free-grazing farming and history of abortion. Vaccination remains the most appropriate control measure in Uganda because brucellosis is endemic and stamping out may be economically too burdensome. Vaccination campaigns, especially focusing on large free-grazing herds in the peri-urban areas, could significantly reduce the prevalence of brucellosis in cattle in the areas and reduce the risk of transmission to humans.

## Methods

### Study sites

The urban and peri-urban areas of Kampala are semi-humid areas with mean annual rainfall of 1261 mm between 1999 and 2005 (standard deviation: 197) [39]. Common production systems are zero-grazing in urban and mixed crop-livestock systems in peri-urban and rural areas. Zero-grazing is also practised in peri-urban areas. Communal grazing, tended by herdsmen, is common in peri-urban areas and rural areas close to the peri-urban areas. A small number of large-scale dairy farms with fenced large grazing yards are also seen in these areas. All these systems were represented in the herds sampled in this study.

Uganda has an administrative system comprising five levels: District (called as Local Council 5), County (LC4), Sub-County (LC3), Parish (LC2) and zone/village (LC1) [40].

**Figure 2:**
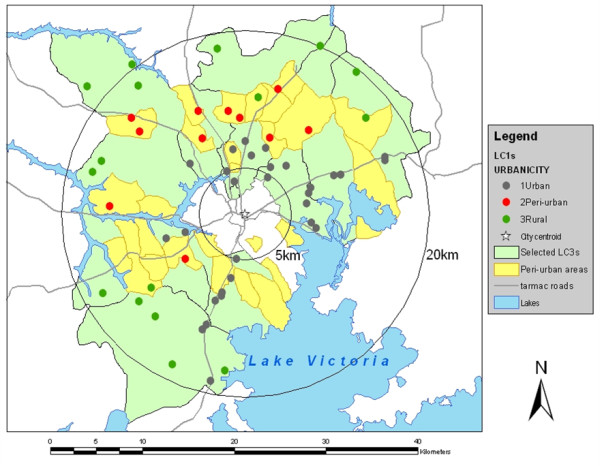
**Selected study sites**. 56 Local Councils I (29 urban, 11 peri-urban and 16 rural LC1s) in the Kampala economic zone were selected for the study.

This study was conducted in 56 cattle-keeping LC1 units in urban (29 LC1s), peri-urban (11 LC1s), and rural areas (16 LC1s) of the Kampala economic zone in Uganda (see Figure 2). Stratified random sampling was used; strata were LC3s (Sub-counties) and sampling units were LC1s. LC3s where more than half of the area is located between five and 20 km from Nakasero, the political and economic centre of Kampala city, were selected for the study. The numbers of LC1s in each stratum were assigned using proportional allocation [41]. Eighty seven LC1s were selected from 790 LC1s in the 10 LC3s. Three LC3s in the central part of Kampala District were excluded from the selection because more than half of the areas were located within 5 km from Nakasero. LC1 leaders and residents were interviewed and the 87 LC1s were classified into urban, peri-urban and rural, based on a decision-tree model as previously described [42]. Of these LC1s, non-residential LC1s (e.g. universities and institutions) and LC1s without cattle were excluded. In the final analysis, 56 LC1s with cattle herds/individuals were selected.

### Ethical statement

This study involves an investigation using interviews with farmers as well as blood sampling from cattle. The study protocol was assessed and approved by the Uganda National Council for Science and Technology (UNCST) on 14th September 2005, with its reference number A 432.

### Multi-stage Sampling framework

#### (1) Sample size of primary sampling units

Primary sampling units (cattle herds) were calculated based on a census of the total number of cattle herds in the selected 56 LC1s, obtained from interviews with LC1 Leaders [42]. WinEpiscope 2.0 [43] was used to calculate sample size for prevalence estimates. The expected herd prevalence was set to 55.6% based on the brucellosis herd prevalence in Mbarara [13], with an accepted error and level of confidence selected as ±5% and 95% respectively. The sampling fraction (24%) that is, the percentage of herds to sample among total herds in the 56 LC1s was calculated by dividing the calculated sample size by total number of herds estimated from above interviews. This sample fraction (24%) was used to determine the sample size of cattle herds in each LC1 at the sampling based on the complete list of cow-keeping farmers prepared by the LC1 Leader in advance.

#### (2) Sample size of secondary sampling units

Milking cows, including cows in the dry period, were selected as secondary sampling units. Bulls, calves and heifers were excluded from the present study because the focus was on cows as potential sources of infected milk. Since milk from different cows is usually mixed at the farm before selling, each farm should be judged as either infected with *Brucella *or not, by determining whether a farm is free from the disease, or has at least one serologically positive cow. To determine the sample size in the field, a card with the sample size of cows for disease detection with the probability of causing error less than 5% in different farm sizes was prepared using FreeCalc version 2 (Australian Veterinary Animal Health Services). Sensitivity and specificity were entered as 95.4% and 99.9% respectively as an imperfect test, buffered antigen plate agglutination test (BPAT) (sensitivity 0.954, specificity 0.977) [44], was initially to be used for the present study complemented by competitive enzyme-linked immunosorbent assay (CELISA, sensitivity 1.000, specificity 0.999) [33]; however CELISA was used for all the samples to increase the accuracy of the estimation. Estimated cattle prevalence at individual animal level for the calculation was selected as 5%, in order to test conservatively that the farm is free from brucellosis - a lower value than that used in other similar studies (central and southern parts of Uganda were found to have 8-16% of herds positive [15], and in Mbarara, herd prevalence was 55.6% [13].

### Herd selection

Sample herds were selected in each LC1 on the basis of random sampling from the list of all cow-keeping farms. Any listed cattle farms that did not have a cow were excluded from the list of cattle herds before herd selection. Prior to sampling, verbal consent was given by each farmer. When permission for sampling or interview was declined, another herd was selected from the listed herds on the basis of random sampling.

### Cattle sampling - Interviews with farmers

Cattle sampling was conducted during October and November 2007. Cattle owners were interviewed for information about their farms and milking cows using a questionnaire. Data from interviews and diagnostic tests were digitized using Microsoft Access (Microsoft Office XP, Redmond, USA). A nine-point body condition score, using half-point increments from 1 to 5 [45], was recorded for all sampled cows.

### Collection of blood samples

Blood was taken from either the jugular or sacral medial vein of cows using 21 gauge needles and disposable 5 ml plastic syringes. Blood was kept in plain vacuum plastic tubes (Vacutainer^®^) and left for 30 minutes to 1 hour at ambient temperature to separate serum from the blood clot. Serum was collected from the Vacutainer using a disposable plastic Pasteur pipette, dispensed to an Eppendorf tube and stored in a cool-box in the field. Eppendorf tubes were then stored in the freezer at -20°C.

### Serological tests

CELISA kits were purchased from the Veterinary Laboratories Agency (Surrey, UK). Kits were sent directly to Uganda, maintaining the cold chain and, immediately after receipt, were kept refrigerated at the Central Laboratory of the Department of Veterinary Medicine, Faculty of Veterinary Medicine, Makerere University, as instructed by the suppliers. CELISA was performed following the manufacturer's protocol at the Molecular Laboratory, Department of Molecular Biology, Faculty of Veterinary Medicine, Makerere University.

### Statistical analysis

Test prevalence was regarded as the true prevalence because sensitivity and specificity of CELISA were 1.000 and 0.999 respectively [33]. As the present study used multi-stage sampling and intra-class correlations at the levels of LC3, LC1 and farm can affect the variance of the estimated prevalence, robust variance estimation [23] was performed using Generalized Estimating Equation (GEE) [46] in statistical software R version 2.13.1. The levels which correlations were controlled were LC3 and LC1 for the herd prevalence, and LC3, LC1 and farm for the animal prevalence. For the animal prevalence estimation, the numbers of cows sampled in the farms were served as offsets to weigh the inter-farm variance and the GEE was performed with Poisson errors.

The geometric mean was used to calculate the mean herd (farm) size because there were some outliers and the data error structures were not Normally distributed.

Within-herd prevalence and the 95% confidence interval, and the relationship between within herd prevalence and herd size (number of cows per farm) were analyzed using a GLM with binomial errors using R.

For risk factors for brucellosis at the animal level, univariate analysis was used. Prevalence ratios were calculated for use of bull for insemination, history of vaccination against brucellosis, and history of abortion using unconditional maximum likelihood estimation for the risk ratio and Fisher exact p-values in EpiTool version 0.5-6 [47] in R. Body condition score was compared between CELISA positive and negative cows using Kruskal-Wallis Rank Sum Test in R. Mean numbers of births and mean age was compared between CELISA positive and negative cows using a One-Way ANOVA after log-transformation with R because the transformation parameter λ (lambda) in Box-Cox transformation [48] statistic was close to zero.

For risk factors for brucellosis at the herd level, univariate analysis was performed. The number of milking cows in a herd- herd size- was compared between sero-positive herds and sero-negative herds using GLM with quasipoisson errors as data were overdispersed. Prevalence ratios were calculated for farming style (free-grazing and movement-restricted farming: tethered and zero-grazing), insemination (use of artificial insemination (AI) or bull), history of vaccination against brucellosis, history of abortion, bought-in sampled cows, and existence of family member or cattle keeper with persistent fever using EpiTools in R. For categorical data: level of urbanisation (urban, peri-urban and rural) and cattle breed, GLMs with binomial errors with the predictors were compared with the GLMs without the predictors by likelihood ratio tests in R.

Factors at the herd level with the p-value less than 0.2 [23] in univariate analysis were investigated further. The factors which do not have collinearity with other factors (correlation coefficient less than 0.9) were fed into a multivariable logistic regression model. Step-wise model simplification was performed checking with a likelihood ratio test. The final model was diagnosed for goodness-of-fit using Hosmer-Lemeshow test [24] in LDdiag [49] in R. Confounding was tested monitoring the change of logit of factors by removing a suspected factor from the model [20], and association between the factors suspected for a confounding was tested where necessary.

## Authors' contributions

Conceived and designed the study: KM EMF CW MCE MT & SCW. Conducted field surveys: KM CW. Analyzed the data: KM. Wrote the paper: KM EMF SCW. All authors read and approved the final manuscript.
